# Ultrasonography of the Vagus Nerve in the Diagnosis of Parkinson's Disease

**DOI:** 10.1155/2020/2627471

**Published:** 2020-03-31

**Authors:** Ovidijus Laucius, Renata Balnytė, Kęstutis Petrikonis, Vaidas Matijošaitis, Neringa Jucevičiūtė, Tadas Vanagas, Vytautas Danielius

**Affiliations:** ^1^Department of Neurology, Lithuanian University of Health Sciences, Eivenių str. 2, LT-50009 Kaunas, Lithuania; ^2^Faculty of Medicine, Medical Academy, Lithuanian University of Health Sciences, Mickevičiaus str. 9, LT 44307 Kaunas, Lithuania

## Abstract

**Background:**

It is currently impossible to diagnose Parkinson's disease (PD) in the premotor phase even though at the time of motor symptom onset the number of already degenerated dopaminergic substantia nigra neurons is considerable. Degeneration of the dorsal nucleus of the vagus nerve (VN) has been reported early in the disease course, and it could lead to impaired function of the VN, resulting in certain nonmotor symptoms of PD. Therefore, we raised a hypothesis that the loss of VN neurons could result in a smaller diameter of the VN among PD patients.

**Methods:**

20 PD patients and 20 age- and gender-matched individuals without any neurodegenerative disease were enrolled in a pilot study. The diameters of the right and left VNs were measured using ultrasonography, their average was calculated, and the narrower VN diameter was noted separately.

**Results:**

No difference was found between the PD and control groups neither in the average VN diameter (mean 1.17; 95% confidence interval (CI) 1.10–1.24 vs. 1.13; 1.07–1.18, mm; *p*=0.353) nor in the narrower VN diameter (mean 1.11; 95% confidence interval (CI) 1.02–1.20 vs. 1.07; 1.02–1.13, mm; *p*=0.421). The narrower VN diameter and the average VN diameter were not able to distinguish between PD patients and controls (area under curve (AUC) = 0.588, 95% CI = 0.408–0.767, and *p*=0.344; and AUC = 0.578, 95% CI = 0.396–0.759, and *p*=0.402).

**Conclusions:**

To conclude, no differences were found in VN diameter between the PD and control groups. Therefore, our data do not support the hypothesis that PD could be associated with a smaller diameter of the VN.

## 1. Introduction

PD is characterized by progressive neuronal loss in the substantia nigra (SN) pars compacta and widespread aggregation of the *α*-synuclein protein that accumulates in Lewy bodies (LB) and Lewy neurites [1]. LB are detected in the SN, the locus coeruleus, the basal nucleus of Meynert, the cerebral cortex [2], and in many other parts of the brain of PD patients, including all cranial nerve nuclei, especially the dorsal motor vagal nucleus and the solitary nuclei [3–5]. Even though a possible neuroprotective role of LB has been suggested in some studies [2], LB have traditionally been considered toxic because the most significant neuronal loss is found in the predilection sites for LB, particularly in the SN and locus coeruleus [2]. The loss of SN dopaminergic neurons results in dopamine depletion that leads to the typical motor symptoms of PD. Even though at the time of their onset up to 66% of SN dopaminergic neurons are already lost [4], it is currently impossible to diagnose PD in the premotor phase. Many potential disease-modifying agents are under research [5], and in case their efficacy is proven for the treatment of PD, treatment should begin when neuronal loss is minimal; therefore, the ultimate goal would be to diagnose PD in the premotor phase; however, due to the unspecificity of the premotor symptoms, further testing is required. Developments in magnetic resonance imaging, positron emission tomography, and single-photon emission tomography raise the possibility of noninvasive diagnostic testing for premotor PD [6]. Nevertheless, other potential premotor PD screening tests that are widely available, easy to use, and less expensive than these imaging methods should be investigated. One of these hypothetical methods is ultrasonography of the vagus nerve (USVN).

The accumulation of alpha-synuclein aggregates in some VN nuclei can be observed even at the earliest stages of PD, and it could result in impaired function of the VN, leading to the development of certain nonmotor symptoms of PD [7–9]. The loss of neurons in vagal nuclei could result in thinning of the VN, detectable by ultrasound. A reduction of the cross-sectional area (CSA) of the VN in patients with PD has been recently shown in two studies [10, 11]. Tsukita et al. reported significantly smaller CSA of the VN on both sides in PD patients (*n* = 21) compared with control subjects (*n* = 21) (right 1.58 vs. 2.35 and left 1.46 vs. 1.91, mm^2^) [10]. Walter et al. measured the VN CSA of 20 PD patients and 20 control subjects and reported similar findings (right 0.64 vs. 1.32 and left 0.69 vs. 1.12, mm^2^) [11]. However, in another study by Fedtke et al., no difference in the CSA between the groups was reported (PD group (*n* = 32): left 2.6, right 2.9 vs. healthy control subjects (*n* = 15): left 2.4, right 2.7, mm^2^) [12]. Despite the similar sample size of these three studies, the heterogeneity of the data is significant. Because the link between PD and the parameters of the VN remains uncertain, we aimed to compare the diameter of the VN between PD patients and healthy control subjects.

## 2. Materials and Methods

### 2.1. Study Design

Following approval by the Lithuanian University of Health Sciences Bioethics Committee, this study was conducted in the Hospital of Lithuanian University of Health Sciences Kaunas Clinics. All 40 patients gave informed consent to participate in the study. 20 PD patients and 20 age- and gender-matched individuals without any neurodegenerative disease were included. The diameters of the right and left VNs were measured using ultrasonography, their average was calculated, and the narrower VN diameter was noted separately. PD patients were also evaluated using the second and third parts of the Movement Disorder Society-Unified Parkinson's Disease Rating Scale (MDS-UPDRS). The stage of PD was evaluated using the modified Hoehn and Yahr scale.

### 2.2. Ultrasonography of the Vagus Nerve (USVN)

**Figure 1:**
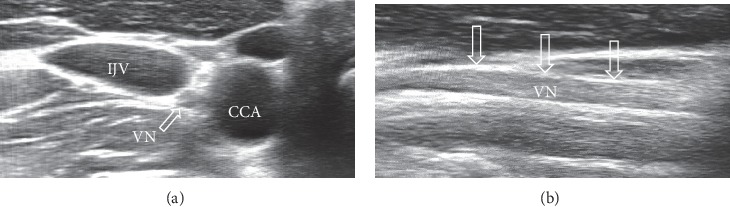
Ultrasonography scan of the vagus nerves in a healthy control subject. (a) Vagus nerve marked with an arrow in an axial image of the neurovascular bundle. (b) Vagus nerve marked with arrows in a sagittal projection. IJV, internal jugular vein; CCA, common carotid artery.

The USVN imaging was performed using a “Voluson 730 Expert” ultrasound device with a linear 6–12  MHz transducer probe, in a depth of ∼3.5 cm. The imaging was performed with the patients resting on their back, with their head bent backward. The probe was placed in the transverse plane several centimetres above the medial ridge of the sternocleidomastoid muscle. The VN can be seen in ultrasound B-mode (Figure 1) near the bifurcation of the carotid artery, dorsal to the internal and common carotid arteries, surrounded by a sheet of connective tissue as a structure that is hypoechogenic in the centre and more hyperechogenic in the periphery [13, 14]. VN diameter was measured in the sagittal plane in millimetres proximal to the carotid bulbus, at the level of the distal end of the common carotid artery.

### 2.3. Statistical Analysis of Data

Statistical analyses were performed using SPSS software package (version 25.0; IBM). Descriptive statistics were calculated. Data normality was assessed using histograms and the Shapiro–Wilk test. Nonparametric tests were used for non-normally distributed variables. The Student's *t* and Mann–Whitney *U* tests were used to evaluate the differences between the groups. The correlation between continuous variables was analysed using Spearman's rank correlation test. Receiver operating characteristic (ROC) curves were used to evaluate the diagnostic performance of the tests. All *p* values are two-sided; The *p* values less than 0.05 were regarded as statistically significant.

## 3. Results

The study sample included 20 PD patients and 20 healthy controls. There were 10 females and 10 males in each group. The clinical characteristics of PD patients are presented in Table 1. The majority of PD patients were mildly or moderately affected.

The age of PD patients and the control subjects did not differ (Table 2) and ranged from 55 to 81 years and from 54 to 80 years, respectively. No differences were found between the PD and control groups neither in the average diameter of the VN nor in the diameter of the narrower VN (Table 2).

In the control group, age did not correlate neither with the average diameter of the VN (*r* = −0.191 and *p*=0.420) nor with the diameter of the narrower VN (*r* = −0.047 and *p*=0.843).

**Figure 2:**
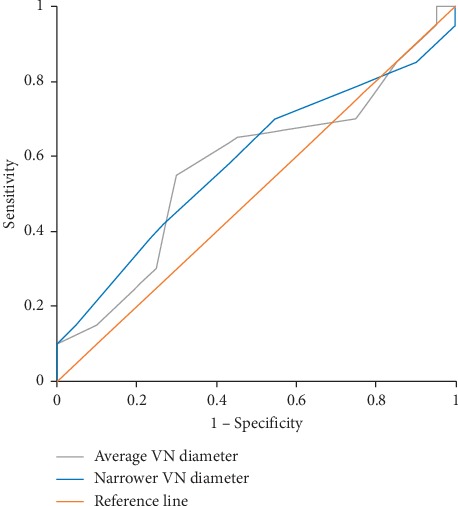
Discriminative abilities of the narrower and average vagus nerve diameters for Parkinson's disease. VN, vagus nerve.

The average VN diameter and the narrower VN diameter showed no statistically significant correlation with age, duration of disease, MDS-UPDRS II, or MDS-UPDRS III in the PD group (data not shown). Both the diameter of the VN on the narrower side and the average diameter on both sides were not able to distinguish between PD patients and healthy subjects (area under curve (AUC) = 0.588, 95% CI = 0.408–0.767, and *p*=0.344; and AUC = 0.578, 95% CI = 0.396–0.759, and *p*=0.402, respectively) (Figure 2).

## 4. Discussion

In this study, we observed no statistically significant difference in the diameter of the VN between PD patients and healthy controls even though VN diameter tended to be slightly greater in the PD group. Our results are in accordance with the ones reported by Fedtke et al. who also observed no significant differences in the VN CSA between controls and the PD group [12]. Our results are contrary to the ones reported in two other studies [10, 11]. A few reasons could be explanatory of the varying results. First, the level chosen for the measurement of the VN was not identical. We measured VN diameter at the level of the distal end of the common carotid artery, and the same approach was chosen by Tsukita et al. [10]. Walter et al. measured VN CSA at the level of the thyroid cartilage [11], and Fedtke et al. did not indicate the exact level chosen for measuring the VN [12]. However, only this difference in methodology could not explain the fact that some studies found significant atrophy of the VN in PD patients, whereas this was not observed in others. Another reason that could explain the varying results is the difference in clinical characteristics of the PD group. However, VN CSA values reported by Walter et al. were a few times lower than the ones shown by Fedtke et al. despite similar age and MDS-UPDRS III score values (30.7 ± 12.4 and 33 ± 12) [11, 12]. The majority of patients were at stage II-III in our study as well as in the studies by Tsukita et al. and Fedtke et al. [10, 12]. Because it seems that the clinical characteristics of patients did not differ so substantially that they could have resulted in such different measurements, the explanation of the heterogeneity of results remains unclear.

Studies exploring the association between VN CSA and age have also reported different results: a decrease in CSA among older subjects was reported in one study [15], whereas another one showed no association [16]. The results of our study were in accordance with the latter ones.

Even though the results of this study are important as now there are two studies that support the association between vagal atrophy and PD and two other studies with opposite results, the limitations of our study should be emphasised. First, only the diameter of the VN rather than both the diameter and the CSA was measured. Second, only one examiner evaluated the diameter of the VN. In addition, this was a single-centre study with a small sample size.

Because of heterogeneous results of all studies examining the association between the VN and PD, further studies should be performed. We suggest performing a larger scale study that would include approximately equal percentages of PD patients in all stages of the disease. In order to ensure the reliability of vagal measurements, all subjects should undergo two ultrasonography evaluations by different physicians and both the diameter and the CSA of the VN should be recorded on both sides at different measurement levels.

## 5. Conclusions

In conclusion, it remains unclear whether PD is associated with structural changes of the VN; therefore, although our results do not indicate the potential value of VN ultrasonography in the diagnosis of PD, future studies are warranted.

## Figures and Tables

**Table 1:** Clinical characteristics of PD patients.

Clinical characteristics		PD patients (*n* = 20)
Duration of PD, years		4.0 (1.0–6.25)

MDS-UPDRS II, points		8.5 (7.0–18.0)

MDS-UPDRS III, points		25.0 (21.3–32.8)

Hoehn and Yahr stages of PD	1	1 (5%)
	1.5	4 (20%)
	2	5 (25%)
	2.5	4 (20%)
	3	4 (20%)
	4	1 (5%)
	5	1 (5%)

Values are given as median (interquartile range) or number (percentage); MDS-UPDRS, Movement Disorder Society-Unified Parkinson's Disease Rating Scale; PD, Parkinson's disease.

**Table 2:** Comparison of age and VN diameter between the PD and control groups.

		PD group (*n* = 20)	Control group (*n* = 20)	*p* value
Age, years	Median	65.5	66.5	0.620
	IQR	58.3–76.0	60.5–76.3	
	95% CI	62.71–71.29	63.99–71.51	

Average VN diameter, mm	Mean	1.17	1.13	0.353
	95% CI	1.10–1.24	1.07–1.18	

Narrower VN diameter, mm	Mean	1.11	1.07	0.421
	95% CI	1.02–1.20	1.02–1.13	

CI, confidence interval; IQR, interquartile range; PD, Parkinson's disease; SD, standard deviation; VN, vagus nerve.

## Data Availability

The underlying data related to this study are available from the corresponding author upon request.
